# Comparative evaluation of gene-set analysis methods

**DOI:** 10.1186/1471-2105-8-431

**Published:** 2007-11-07

**Authors:** Qi Liu, Irina Dinu, Adeniyi J Adewale, John D Potter, Yutaka Yasui

**Affiliations:** 1 School of Public Health, University of Alberta, Edmonton, Alberta, T6G2G3, Canada; 2 Division of Public Health Sciences, Fred Hutchinson Cancer Research Center, Seattle, Washington, 98109, USA

## Abstract

**Background:**

Multiple data-analytic methods have been proposed for evaluating gene-expression levels in specific biological pathways, assessing differential expression associated with a binary phenotype. Following Goeman and Bühlmann's recent review, we compared statistical performance of three methods, namely Global Test, ANCOVA Global Test, and SAM-GS, that test "self-contained null hypotheses" Via. subject sampling. The three methods were compared based on a simulation experiment and analyses of three real-world microarray datasets.

**Results:**

In the simulation experiment, we found that the use of the asymptotic distribution in the two Global Tests leads to a statistical test with an incorrect size. Specifically, p-values calculated by the scaled *χ*^2 ^distribution of Global Test and the asymptotic distribution of ANCOVA Global Test are too liberal, while the asymptotic distribution with a quadratic form of the Global Test results in p-values that are too conservative. The two Global Tests with permutation-based inference, however, gave a correct size. While the three methods showed similar power using permutation inference after a proper standardization of gene expression data, SAM-GS showed slightly higher power than the Global Tests. In the analysis of a real-world microarray dataset, the two Global Tests gave markedly different results, compared to SAM-GS, in identifying pathways whose gene expressions are associated with *p53 *mutation in cancer cell lines. A proper standardization of gene expression variances is necessary for the two Global Tests in order to produce biologically sensible results. After the standardization, the three methods gave very similar biologically-sensible results, with slightly higher statistical significance given by SAM-GS. The three methods gave similar patterns of results in the analysis of the other two microarray datasets.

**Conclusion:**

An appropriate standardization makes the performance of all three methods similar, given the use of permutation-based inference. SAM-GS tends to have slightly higher power in the lower *α*-level region (i.e. gene sets that are of the greatest interest). Global Test and ANCOVA Global Test have the important advantage of being able to analyze continuous and survival phenotypes and to adjust for covariates. A free Microsoft Excel Add-In to perform SAM-GS is available from .

## Background

Some microarray-based gene expression analyses such as Significance Analysis of Microarray (SAM) [1] aim to discover *individual genes *whose expression levels are associated with a phenotype of interest. Such *individual-gene analyses *can be enhanced by utilizing existing knowledge of biological pathways, or sets of individual genes (hereafter referred to as "gene sets"), that are linked via. related biological functions. *Gene-set analyses *aim to discover *gene sets *the expression of which is associated with a phenotype of interest.

Many *gene-set analysis *methods have been proposed previously. For example, Mootha *et al. *[2] proposed Gene Set Enrichment Analysis (GSEA), which uses the Kolmogorov-Smirnov statistic to measure the degree of differential gene expression in a gene set by a binary phenotype (see also [3]). Goeman *et al. *[4] presented Global Test, modeling differential gene expression by means of random-effects logistic regression models. Goeman *et al. *[5] also extended their methods to continuous and survival outcomes. Mansmann and Meister [6] proposed ANCOVA Global Test, which is similar to Global Test but having the roles of phenotype and genes exchanged in regression models. Mansmann and Meister [6] pointed out that their ANCOVA Global Test outperformed Global Test, especially in cases where the asymptotic distribution of Global Test cannot be used. Dinu *et al. *[7] discussed some critical problems of GSEA as a method for gene-set analysis and proposed an alternative method called SAM-GS, an extension of SAM to gene-set analysis. Goeman and Bühlmann [8] provided an excellent review of the methods, discussing important methodological questions of gene-set analysis, and summarized the methodological principles behind the existing methods. An important contribution of their review was the distinction between testing "self-contained null hypotheses" via. subject sampling and testing "competitive null hypotheses" via. gene sampling. They argue, and we agree, that the framework of the competitive hypothesis testing via. gene sampling is subject to serious errors in calculating and interpreting statistical significance of gene sets, because of its implicit or explicit untenable assumption of probabilistic independence across genes.

Although Global Test, ANCOVA Global Test, and SAM-GS each test a self-contained hypothesis on the association of expression patterns across a gene set with a phenotype of interest in a statistically appropriate manner, it is unclear how the three methods compare on performance in detecting underlying associations. In this paper, we compare the performance of the three methods via. simulation and real-world microarray data analyses, both statistically and biologically.

## Results

### Simulation experiment

Our first evaluation of the three methods used a simulation study, similar to that of Mansmann and Meister [6] with some modifications that make the simulated data more realistic, and evaluated the size and power of the three hypothesis tests. Gene-set analysis was performed for both the original and "z-score standardized" simulated datasets so that the effects of standardization on the three tests' performance can be assessed. The z-score standardization was motivated by that fact that gene-expression variances can vary greatly across genes, even after a normalization, which could influence gene-set analysis. In the z-score standardized datasets, gene expression was standardized using the following equation:

$$x_{jk}^{'}=\frac{x_{jk}-{\bar{x}}_{j}}{s_{j}}$$

where *x*_*jk *_is the gene expression for gene *j *in sample *k*, ${\bar{x}}_{j}$ and *s*_*j *_are the sample mean and standard deviation of gene *j *expression using all samples, respectively. All simulation analyses compared the mean expression of a gene-set of interest between two groups, each with a sample of 10 observations.

First, we checked the size of the three tests, before and after the standardization, according to the following three scenarios of no differential expression between two groups: (1) randomly generate expression of 100 genes for the two groups from a multivariate normal distribution (MVN) with a mean vector ***μ ***and a diagonal variance-covariance matrix Σ, where the 100 elements of ***μ ***and the 100 diagonal elements of Σ were randomly generated as 100 independently-and-identically-distributed (i.i.d.) uniform random variables in (0,10) and 100 i.i.d. uniform random variables in (0.1, 10), respectively (i.e., no gene was differentially expressed between the two groups and expression was uncorrelated among the 100 genes); (2) exactly same as (1) except the variance-covariance matrix Σ of the MVN being changed to have a correlation of 0.5 between all pairs of the first 20 genes and also between all pairs of the second 20 genes; (3) exactly same as (2) with the correlation value changed from 0.5 to 0.9.

Second, we estimated the power of the three tests, before and after the standardization, by randomly generating a gene set of size 100, using the exactly same simulation set-up of the size-evaluation (2) above, but allowing the first 40 genes being differentially expressed. The mean expression of the 40 differentially expressed genes was randomly generated from Uniform(0,10) as in the size-evaluation (2), but was subsequently modified by an addition and a subtraction of a constant *γ*, as in Mansmann and Meister [6], such that mean vectors ***μ***_*i*_'s for the two groups (*i *= 1, 2) differ by 2*γ*, $\mu_{1j}-\mu_{2j}={\left(-1\right)}^{I_{j>20}}2\gamma$, for *j *= 1,..., 40. We considered a range of *γ *from 0 to 2 with an increment of 0.1. The 40 differentially expressed genes were set to have a correlation of 0.5, as in the size-evaluation (2), but no correlation and a correlation of 0.9 were also considered.

In the comparison of size across the three tests, the size was estimated by the observed proportion of replications with a p-value smaller than the correct size *α*. By definition, under the null hypothesis, a proportion *α *of the replications of an experiment is expected to yield a p-value smaller than *α*. In order to assess the size, we ran 5000 replications and used *α *= 0.05. For each permutation-based p-value, 1000 random permutations were carried out.

In the comparison of power across the three tests, the power was estimated by the observed proportion of the replications of an experiment in which the null hypothesis was correctly rejected. Given the fixed numbers of samples and genes with the fixed correlation structure in the simulation experiment, a larger effect size *γ *leads to higher power for a given *α*-level. In estimating the power, we ran 1000 replications of an experiment for each *γ *value. We considered *α *at 0.05, 0.01, 0.005, 0.0025, and 0.001. For obtaining a permutation-based p-value, 1000 random permutations were carried out.

The empirical Type I error rates of SAM-GS and the two Global Tests with permutations were almost right on the target of the nominal value of 0.05, before and after the standardization, for all three scenarios considered for the evaluation of size (Table 1). Type I error rates of Global Test with the scaled *χ*^2 ^null distribution and Global Test with the asymptotic null distribution with a quadratic form deviated noticeably from the nominal size, being too liberal with the scaled *χ*^2 ^and too conservative with the asymptotic distribution (non *χ*^2 ^distributed quadratic form) as shown in Table 1. As the correlation among the 40 genes increased, the Type I error rates of Global Test with the scaled *χ*^2 ^null distribution and the Global Test with the asymptotic null distribution with a quadratic form generally moved towards the nominal size of 0.05. Type I error rates of ANCOVA Global Test with the asymptotic distribution also deviated noticeably from the nominal size: 0.0692, 0.1034 and 0.0898 before the standardization and 0.037, 0.0848 and 0.0792 after the standardization, for *r *= 0, 0.5, and 0.9, respectively. Hereafter, therefore, the p-values for Global Test and ANCOVA Global Test are calculated based on permutations. We also estimated the size of the three tests using 25 samples, instead of 10 samples, in each group, and observed similar patterns. As the sample size increased, the Type I error rates of the two Global Tests by using the asymptotic distributions moved towards to the nominal level of 0.05.

**Table 1:** Assessment of type I error probabilities

			**10 vs. 10 samples**			**25 vs. 25 samples**		
			
	**Methods**	**Type of inference**	**0**	**0.5**	**0.9**	**0**	**0.5**	**0.9**
**Before standardization**	Global Test	The scaled *χ*^2^	0.0982	0.0778	0.0722	0.0696	0.0700	0.0686
		Asymptotic	0.0006	0.0128	0.0298	0.0090	0.0328	0.0442
		Permutation	0.0496	0.0434	0.0464	0.0534	0.0554	0.0556
	ANCOVA Global Test	Asymptotic	0.0692	0.1034	0.0898	0.0576	0.0840	0.0736
		Permutation	0.0482	0.0462	0.0458	0.0526	0.0552	0.0562
	SAM-GS	Permutation	0.0498	0.0462	0.0478	0.0514	0.0518	0.0556
**After standardization**	Global Test	The scaled *χ*^2^	0.1090	0.0844	0.0736	0.0734	0.0702	0.0698
		Asymptotic	<0.0001	0.0094	0.0276	0.0036	0.0320	0.0424
		Permutation	0.0524	0.0464	0.0458	0.0524	0.0528	0.0530
	ANCOVA Global Test	Asymptotic	0.0372	0.0848	0.0792	0.0474	0.0838	0.0730
		Permutation	0.0532	0.0462	0.0466	0.0544	0.0542	0.0544
	SAM-GS	Permutation	0.0522	0.0468	0.0470	0.0526	0.0540	0.0542

**Figure 1:**
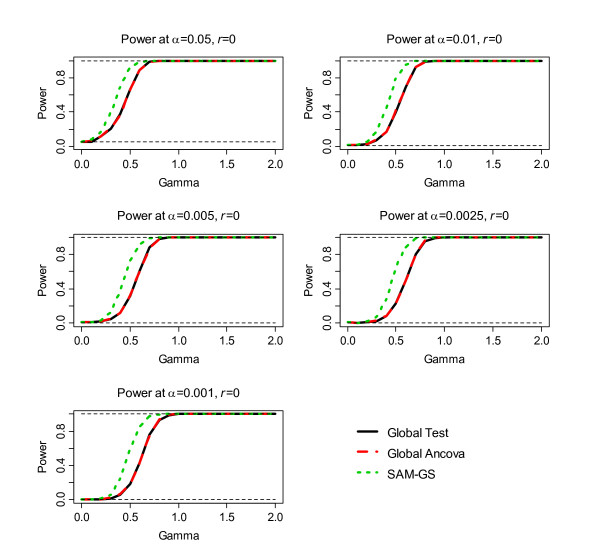
The results of the simulation experiment, evaluating power of the three tests before the standardization, for correlation of 0 among 40 genes.

**Figure 2:**
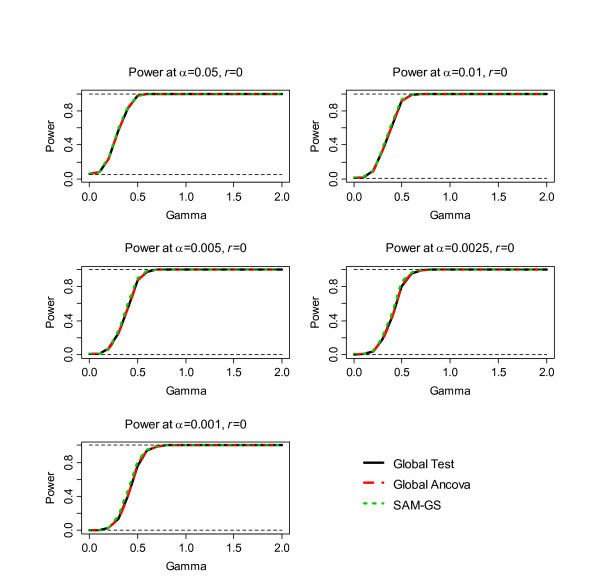
The results of the simulation experiment, evaluating power of the three tests after the standardization, for correlation of 0 among 40 genes.

**Figure 3:**
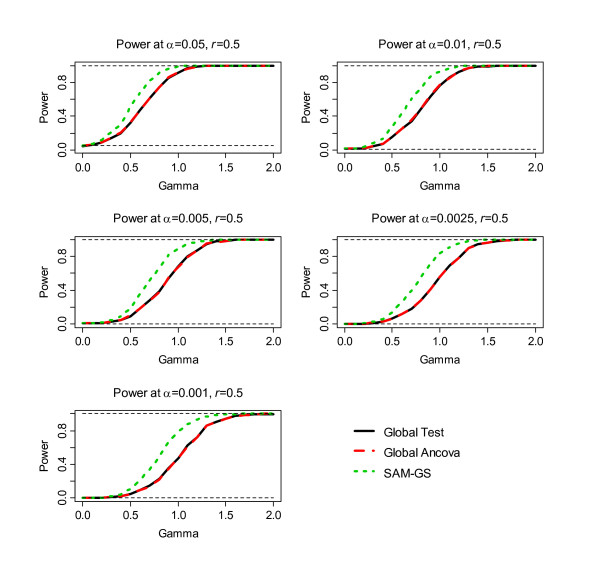
The results of the simulation experiment, evaluating power of the three tests before the standardization, for correlation of 0.5 among 40 genes.

**Figure 4:**
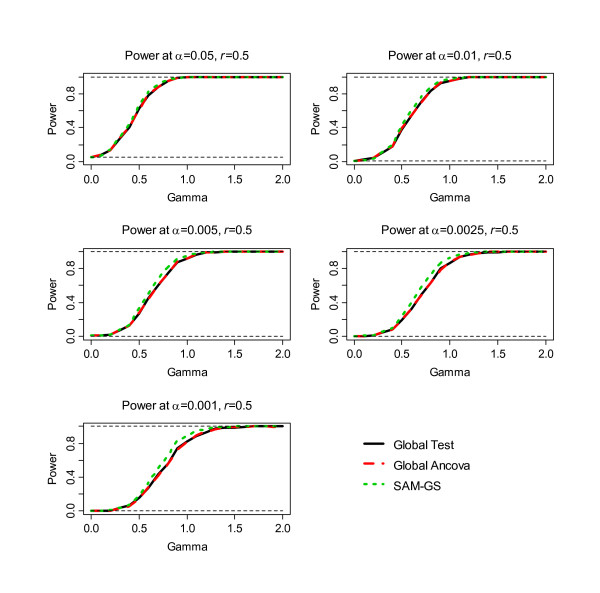
The results of the simulation experiment, evaluating power of the three tests after the standardization, for correlation of 0.5 among 40 genes.

**Figure 5:**
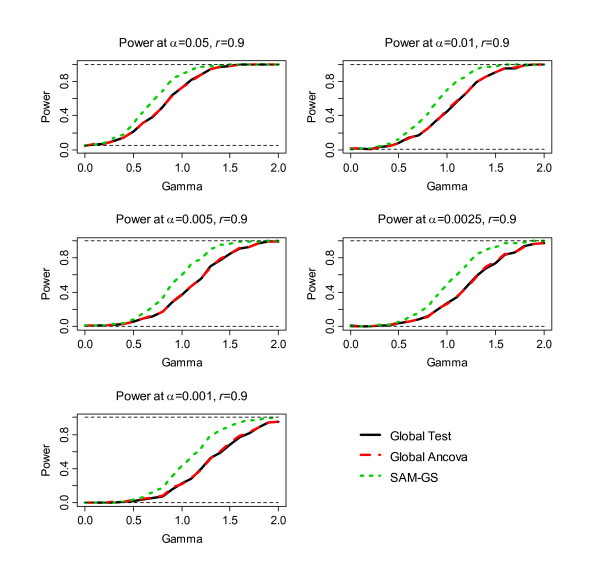
The results of the simulation experiment, evaluating power of the three tests before the standardization, for correlation of 0.9 among 40 genes.

The second step of the simulation was to assess power, the results of which are shown in Figure 1, 2, 3, 4, 5, 6. Before the standardization, SAM-GS showed higher power than the Global Tests at *α *= 0.05, with increasing power differences with decreasing *α *levels. This pattern was observed regardless of the correlation level in the 40 differentially-expressed genes (correlation of 0, 0.5, or 0.9). After the standardization, the performances of these three methods became closer: SAM-GS showed slightly higher power than the Global tests with increasing power difference with decreasing *α *levels.

**Figure 6:**
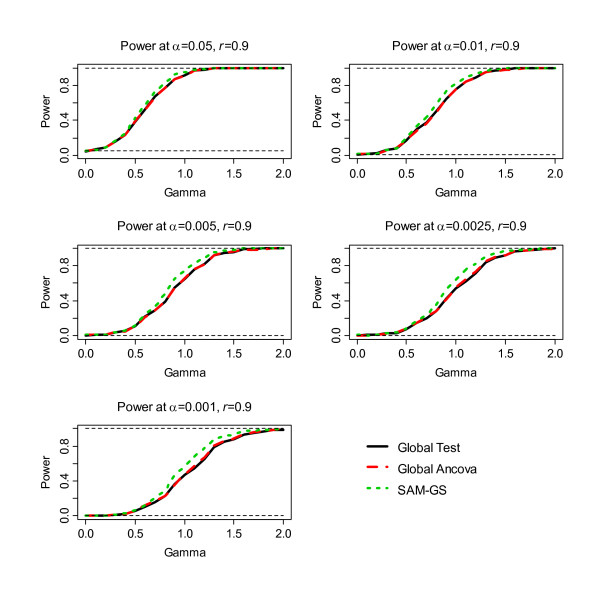
The results of the simulation experiment, evaluating power of the three tests after the standardization, for correlation of 0.9 among 40 genes.

### Real-world data analyses

Our next evaluation of the performance of the three methods used biologically, a priori defined gene sets and three microarray datasets considered in Subramanian *et al*. [3], download from GSEA web-page, [9]: 17 *p53 *wild-type vs. 33 *p53 *mutant cancer cell lines; 15 male vs. 17 female lymphoblastoid cells; 24 acute lymphoid leukemia (ALL) vs. 24 acute myeloid leukemia (AML) cells. For pathways/gene sets, we used Subramanian *et al*.'s gene-set subcatalogs C1 and C2 from the same web-address above on "Molecular Signature Database." The C1 catalog includes gene sets corresponding to human chromosomes and cytogenetic bands, while the C2 catalog includes gene sets that are involved in specific metabolic signaling pathways [3]. In Subramanian *et al*., Catalog C1 included 24 sets, one for each of the 24 human chromosomes, and 295 sets corresponding to the cytogenetic bands; Catalog C2 consisted of 472 sets containing gene sets reported in manually curated databases and 50 sets containing genes reported in various experimental papers. Following Subramanian *et al*. [3], we restricted the set size to be between 15 and 500, resulting in 308 pathways to be examined.

We compared the performance of the three methods before and after the standardization by listing the gene sets which had a p-value ≤ 0.001 by any of the three methods.

Table 2 shows the associations of biologically-defined gene sets with the phenotype, assessed by Global Test, ANCOVA Global Test, and SAM-GS, in the analysis of gene expression differences between *p53 *wild-type vs. mutant cancer cell lines. Gene sets with a p-value ≤ 0.001 by any of the three methods are listed in Table 2. Before the standardization, SAM-GS identified 16 gene sets with a p-value ≤ 0.001, while Global Test and ANCOVA Global Test identified three and one gene sets, respectively, with a p-value ≤ 0.001 (Table 2). Two of these three sets were among the 16 sets identified by SAM-GS. The third set was CR_DEATH which had a p-value = 0.008 from SAM-GS. Among the 17 gene sets listed in Table 2, seven involve *p53 *directly as a gene-set member. Furthermore, five gene sets directly involve the extrinsic and intrinsic apoptosis pathways, and three involve the cell-cycle machinery; each of these eight gene sets, then, is in a direct, well-established relationship with aspects of *p53 *signaling. The remaining two gene sets have plausible, if less well established, links with *p53 *[7]. The disagreement between results of SAM-GS and the two Global tests was considerable before standardization. Although 16 of the 17 gene sets in Table 2 had a SAM-GS p-value ≤ 0.001, 7 had p-values larger than 0.1 by the two Global tests. For example, SAM-GS identified the gene set *p53*hypoxia pathway as a significant set with a p-value < 0.001, which seems biologically appropriate, yet the Global Test and the ANCOVA Global Test gave p-values greater than 0.6.

**Table 2:** Gene sets in the *p53 *dataset with P-value ≤ 0.001 by any of the three methods

**Gene Set**	**Before standardization**			**After standardization**			**VSN**		
	
	**Global**	**Ancova**	**SAM-GS**	**Global**	**Ancova**	**SAM-GS**	**Global**	**Ancova**	**SAM-GS**
ATM Pathway*	<0.001	<0.001	<0.001	<0.001	0.002	<0.001	0.001	0.001	<0.001
BAD Pathway**	<0.001	0.007	<0.001	<0.001	<0.001	<0.001	0.004	0.004	<0.001
Calcineurin Pathway$	0.068	0.084	<0.001	0.007	0.002	<0.001	0.004	0.005	0.011
Cell cycle regulator†	0.021	0.017	<0.001	0.002	0.001	<0.001	0.002	<0.001	0.003
Hsp27Pathway**	0.047	0.044	<0.001	<0.001	0.001	<0.001	0.011	0.005	<0.001
Mitochondria pathway**	0.002	0.002	<0.001	0.007	0.007	<0.001	0.013	0.006	<0.001
*p53 *signaling pathway*	0.112	0.101	<0.001	0.003	0.003	0.001	0.006	0.005	0.006
*p53*_UP*	0.003	0.004	<0.001	<0.001	<0.001	<0.001	0.019	0.018	<0.001
*p53*hypoxiaPathway*	0.626	0.622	<0.001	<0.001	<0.001	<0.001	0.044	0.041	<0.001
*p53*Pathway*	0.142	0.150	<0.001	<0.001	<0.001	<0.001	<0.001	0.001	<0.001
Raccycd Pathway†	0.177	0.181	<0.001	0.001	<0.001	<0.001	0.004	0.009	0.006
Radiation_sensitivity*	0.119	0.135	<0.001	<0.001	<0.001	<0.001	0.014	0.020	<0.001
SA_TRKA_RECEPTOR‡	0.254	0.252	<0.001	0.001	<0.001	<0.001	0.004	0.001	0.006
bcl2family & reg. network**	0.102	0.100	0.001	0.001	0.005	<0.001	0.010	0.014	0.001
Cell cycle arrest†	0.099	0.099	0.001	0.027	0.018	0.005	0.003	0.005	0.007
Ceramide Pathway**	0.002	0.006	0.001	0.004	0.004	<0.001	0.001	0.001	<0.001
CR_DEATH*	0.001	0.004	0.008	0.029	0.017	0.004	0.143	0.108	0.005

* pathway member

** apoptosis

$ *p53*-induced proline oxidase mediates apoptosis via a calcineurin-dependent pathway

† cell cycle

‡ integrated negative feedback loop between Akt and *p53*

**Figure 7:**
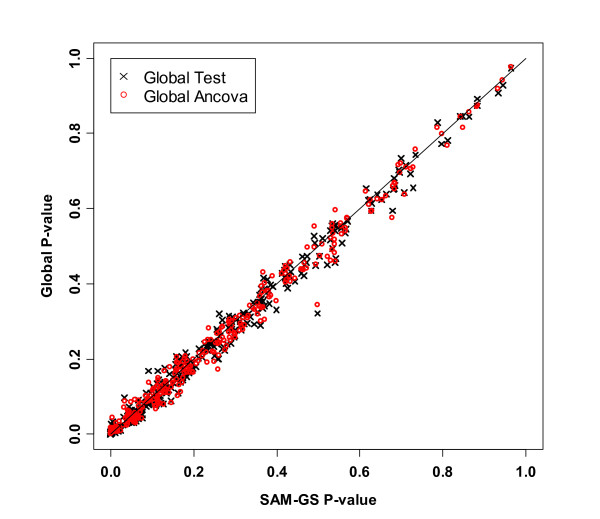
P-values of 308 gene sets in the *p53 *data analysis: p-values of Global Test and ANCOVA Global Test after standardization vs. SAM-GS p-values before the standardization. The line indicates equal p-values between SAM-GS and Global Tests.

**Figure 8:**
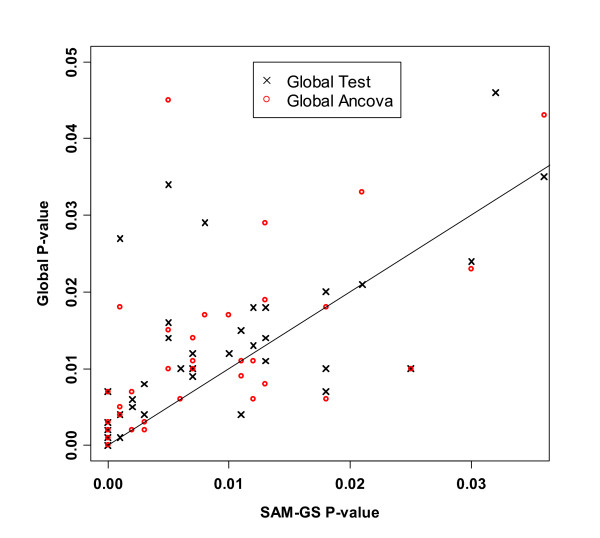
Lowest P-values in the *p53 *data analysis: p-values of Global Test and ANCOVA Global Test after standardization vs. SAM-GS p-values before the standardization. The line indicates equal p-values between SAM-GS and Global Tests.

We then compared the three methods incorporating the z-score standardization. For SAM-GS, the p-values before and after the standardization were highly consistent, and, therefore, we used the results of SAM-GS before the standardization for the comparisons with the other two methods. For Global Test and ANCOVA Global Test, p-values changed appreciably. Notably, p-values of Global Test and ANCOVA Global Test after the standardization agreed closely with those of SAM-GS (Table 2, Figure 7). For example, the p-values of *p53*hypoxia pathway changed from 0.626 to <0.001 for Global Test and from 0.622 to <0.001 for ANCOVA Global Test. Although the p-values of the three methods agreed with each other after the standardization, the p-values from SAM-GS tended to be smaller than those from Global Test and ANCOVA Global Test, in the lower range of p-values (gene sets that are of the greatest interest) (Table 2, Figures 7 and 8): this is consistent with the power-comparison simulation in which SAM-GS showed slightly higher power than the Global tests at small *α *levels, even after the standardization.

The same pattern was found in the analyses of the male-vs.-female lymphoblastoid dataset and the ALL-vs.-AML dataset (See Figures S1, S2, S3 and S4 in Additional file 1, comparing the results from the three methods). Before the standardization, p-values from Global Test and ANCOVA Global Test differed greatly from p-values from SAM-GS. The p-values of Global Test and ANCOVA Global Test changed markedly after the standardization and were very close to those of SAM-GS. After the standardization, in the male-vs.-female analysis, 21 gene sets had a p-value < 0.15 by one or more of the methods; 17 of these had a SAM-GS p-value smaller than, or equal to, those of Global Test and ANCOVA Global Test. In the ALL-vs.-AML analysis, all sets were statistically significant with p-values < 0.001 by all three tests: which is unlikely to be of any biological significance.

**Figure 9:**
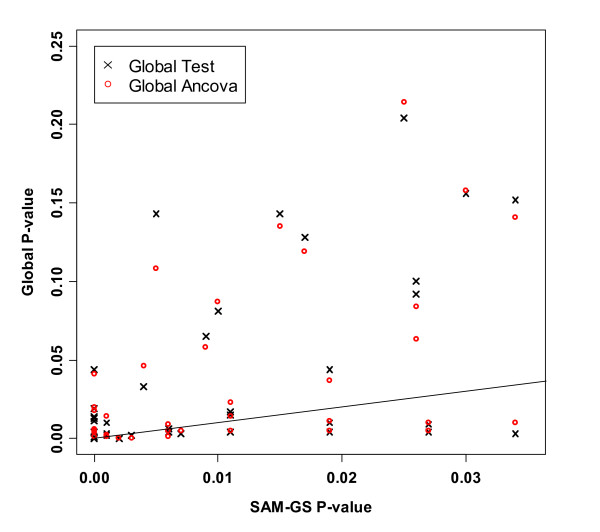
Lowest P-values in the *p53 *data analysis: p-values of Global Test and ANCOVA Global Test after the VSN normalization vs. SAM-GS p-values after the VSN normalization. The line indicates equal p-values between SAM-GS and Global Tests.

In the User Guides for Global Test and ANCOVA Global Test, Variance Stabilization (VSN) was used to normalize the data [10,11]. We also assessed the performance of the three methods on the *p53 *dataset, male vs. female dataset, and the ALL/AML dataset using VSN. The results for the *p53 *dataset are shown in Table 2 and Figure 9. When VSN was used for the normalization of the data, we observed: (1) p-values of Global Test and ANCOVA Global Test became similar to those of SAM-GS, but not as close as the p-values after the z-score standardization; and (2) in the lower range of p-values, the p-values for SAM-GS tended to be smaller than those of Global Test and ANCOVA Global Test, (Table 2, Figure 9).

## Discussion

From the simulation results, we suggest that, when Global Test and ANCOVA Global Test are used for the analysis of microarray data, permutations should always be used for the calculation of statistical significance. In the documentation included with the Global Test R package, Goeman *et al. *noted that the asymptotic distribution with a quadratic form is the recommended method for large sample sizes and it can be *slightly *conservative for small samples. In our simulation study, we used 10 and 25 samples for each of the two groups. In each situation, the asymptotic method with a quadratic form gave conservative p-values, although the difference between asymptotic and permutation-based methods did decrease when the sample size increased. Goeman *et al. *also noted that the scaled *χ*^2 ^method can be *slightly *anti-conservative, especially for large gene sets. Our simulation study showed that the scaled *χ*^2 ^method can be markedly anti-conservative. This is in accord with the manual of Global Test, which recommends against using the scaled *χ*^2 ^approximation.

We found that performance of the two Global Tests changed greatly before and after standardization, but SAM-GS performance remained unchanged. This can be explained by: (1) the invariance of *t*-test statistics under shifting and rescaling of data, that is relevant to SAM-GS; (2) ANCOVA's explicit assumption that all genes in the set to have an equal variance, a violation of which would clearly affect the performance of ANCOVA Global Test; and (3) Global test's assumption that the regression coefficients come from the same normal distribution, an assumption that is met by the standardization of gene expression. Therefore, some sort of standardization that makes the variances of gene expression similar across genes is needed before using Global Test and ANCOVA Global Test. SAM-GS employs a constant in the denominator of its *t*-like test statistic to address the small variability in some of the gene expression measurements and, thus, effectively standardizes expression across genes; neither Global Test nor the ANCOVA Global Test addresses this characteristic of microarray data. Both Goeman *et al. *[4] and Mansmann and Meister [6] have stated that an appropriate normalization is important. Note that not many normalization methods would standardize the expression across genes. It is only after applying z-score standardization (1) or the VSN normalization, that the results of the three methods became congruent. The similarity between Global Test and Global ANCOVA Test has already been commented upon in [6]. The similarity between SAM-GS and Global Test may be inferred from the construction of the latter as a weighted sum of squared transformed t-statistics [12], which is similar to the SAM-GS test statistic.

It should be noted that Global Test allows four different types of phenotype variables: binary; multi-class; continuous; and survival. ANCOVA Global Test allows binary, multi-class, and continuous phenotypes. The ability to handle different classes of phenotypes is a very important advantage of Global Test and ANCOVA Global Test over SAM-GS. It is also possible to use Global Test and ANCOVA Global Test while adjusting for covariates (e.g., potential confounders). If covariates are incorporated, the two tests assess whether the gene-expression profile has an independent association with the phenotype that is above and beyond what is explained by the covariates. The ability to adjust for covariates is another important advantage of Global Test and ANCOVA Global Test over SAM-GS.

We focused on p-values in this paper because we were comparing the three methods that test "self-contained null hypotheses" via. subject sampling. To account for multiple comparisons when multiple gene sets are tested, one might consider False Discovery Rate (FDR) instead of Type I error probability. For example, SAM uses a q-value, an upper limit of the FDR, for each gene, which could be extended here to each gene-set using the method of Storey [13]. The q-values of the 17 gene sets listed in Table 2 are displayed in Additional file 2.

We have considered, but did not report detailed comparison results of two other methods, Tian *et al*. [14] and Tomfohr *et al. *[15], that test self-contained hypotheses via. subject sampling, in addition to the three methods we highlighted above. Tian *et al. *[14] tests the significance of a gene set by taking the mean of t-values of genes in the gene set as a test statistic and evaluating its significance by a permutation test. Tomfohr *et al. *[15] reduces the gene set's expression into a single summary value by taking the first principal component of expressions of genes in the gene set and performs a permutation-based t-test of the single summary. The two methods gave appreciably different results when compared to Global Test and ANCOVA Global Test, and SAM-GS. Of the 17 gene sets in Table 2 for the *p53 *analysis, for instance, Tian *et al*. and Tomfohr *et al. *identified only eight and one gene sets, respectively, with p-value < 0.10: the ATM pathway, for example, was identified by Global Test, ANCOVA Global Test, and SAM-GS with p-value ≤ 0.001, while the methods of Tian *et al*. and Tomfohr *et al. *gave p-value = 0.61 and 0.99, respectively. The main reasons for their large discrepancies from the results of the three highlighted methods are as follows. Tian *et al. *sums up the t-values for all the genes in a gene set, which will result in cancellation of large positive t-values and large negative t-values. Among the 11 up-regulated and 8 down-regulated genes in the ATM pathway, for example, two up-regulated genes had large positive t-values (about 2 or greater) and three down-regulated genes had large negative t-values (about – 2 or smaller): these large positive and negative t-values cancel each other when summing up all t-values in the Tian *et al*. test statistic, leading to reduced power for detecting gene sets that contain both significantly up-regulated genes and significantly down-regulated genes. The method of Tomfohr *et al. *summarizes the |*S*|-dimension gene-expression vector of genes in the gene set *S *by the first principal component without considering the phenotype: if the direction of the first principal component does not correspond to the direction that separates the two phenotypes, their method does not capture the differential expressions even when they exist, leading to markedly reduced power.

Although we focused on the comparison of the "self-contained null hypothesis" approaches, it is also of methodological interest to see how "competitive null hypothesis" approaches compare. We, therefore, applied three "competitive null hypothesis" approaches to the analysis of the *p53 *dataset: Gene Set Enrichment Analysis (GSEA) [2]; the Significance Analysis of Function and Expression (SAFE) [16]; and Fisher's exact test [17]. The results are shown in Additional file 3. The results from the three "competitive null hypothesis" approaches were greatly different from those of SAM-GS and the Global Tests. Most of the gene sets identified as being significantly associated with the *p53 *mutation by SAM-GS and Global Tests were not identified as such by the three "competitive null hypothesis" approaches. The only gene set additionally identified as being significantly associated with the *p53 *mutation (with p < 0.001) was HUMAN_CD34_ENRICHED_TF_JP: for this gene set, the Fisher's exact test p-value was < 0.001, but all the other five methods gave p-values > 0.37. Known biological functions of *p53 *are clearly more consistent with the results of the "self-contained null hypothesis" approaches. The differences observed between "self-contained null hypothesis" and "competitive null hypothesis" approaches can be attributable, at least partly, to the fact that the significance of a gene set depends only on the genes in the set under the "self-contained null hypothesis" testing, while, under the "competitive null hypothesis" testing, the significance of a gene set depends not only on the genes in the set but also on all the other genes in the array.

In summary, the primary advantage of SAM-GS may be the slightly higher power in the low *α*-level region that is of highest scientific interest, whereas, despite the need for appropriate standardization, Global Test and the ANCOVA Global Test can be used for a variety of phenotypes and incorporate covariates in the analysis.

## Conclusion

In conclusion, Global Test and ANCOVA Global Test require appropriate standardization of gene expression measurements across genes for proper performance. Standardization of these two methods and the use of permutation inference make the performance of all three methods similar, with a slight power advantage in SAM-GS. Global Test and the ANCOVA Global Test can be used for a variety of phenotypes and incorporate covariates in the analysis.

## Methods

In this section, we describe the three gene-set analysis methods. The phenotype of interest is assumed to be binary.

### 1) Global Test

The Global Test is based on a regression model that predicts response from the gene expression measurements of a gene set [4]. Generalized linear models are used to model the dependency of response *Y *(an *n *× 1 vector) on gene expression measurements *X *(an *n *× *m *matrix) of a set of *m *genes on *n *samples:

$$\begin{array}{cc} h\left(E(Y_{i}|\beta )\right)=\alpha +\sum_{j=1}^{m}x_{ij}\beta_{j}, & i=1,2,...,n, \end{array}$$

where *h *denotes the link function and *α *and *β*'s are parameters. If the genes are not differentially expressed, the regression coefficients (*β*'s) should be zero. Under an assumption that all regression coefficients are sampled from a common distribution with mean zero and variance *τ*^2^, the null hypothesis of no differential gene-expression is reduced to *τ*^2 ^= 0. Using the notation *r*_*i *_= Σ_*j*_*x*_*ij*_*β*_*j*_, the model simplifies to a random-effects model: *E*(*Y*_*i*_|*r*_*i*_) = *h*^-1 ^(*α *+ *r*_*i*_). The null hypothesis can then be tested, based on a score test statistic discussed in Le Cessie and Van Houwelingen[18] and Houwing-Duistermaat *et al. *[19]:

$$Q=\frac{{\left(Y-\mu \right)}^{'}R(Y-\mu )}{\mu_{2}},$$

where *R *= (1/*m*)*XX'*, *μ *= *h*^-1^(*α*), and *μ*_2 _is the second central moment of *Y *under the null hypothesis. It can be shown that *Q *is asymptotically normally distributed (a quadratic form which is non-negative). However, when the sample size is small, a better approximation to the distribution of *Q *is a scaled *χ*^2 ^distribution. The p-value can, therefore, be calculated based on an approximate distribution of the test statistic, i.e., the asymptotic distribution with a non-chi-squared distributed quadratic form or the scaled *χ*^2 ^distribution, or permutations of samples.

### 2) ANCOVA Global Test

The null hypothesis of the Global Test is in the form of P(Y|X) = P(Y). The ANCOVA Global Test changes the roles of gene expression pattern X and phenotype Y, and the null hypothesis becomes P(X|Y = 1) = P(X|Y = 2), or, for each gene *j *in a gene set of interest, *μ*_1*j *_= *μ*_2*j*_, where *μ*_*ij *_is the mean expression of gene *j *in phenotype group *i*, *i *= 1,2. A linear model of the form, *μ*_*ij *_= *μ *+ *α*_*i *_+ *β*_*j *_+ *γ*_*ij*_, with group effects *α*, gene effects *β*, and the gene-group interaction *γ*, is then used to test the null hypothesis. The conditions Σ*α*_*i *_= Σ*β*_*j *_= Σ_*i*_*γ*_*ij *_= Σ_*j*_*γ*_*ij *_= 0 ensure identifiability of the parameters. The null hypothesis under the parameterization of the linear model is H_0_: *α*_*i *_= *γ*_*ij *_= 0. The test statistic is the F-test statistic for linear models: $F=\{(SSR_{{\text{H}}_{\text{0}}}-SSR_{{\text{H}}_{\text{1}}})/(df_{{\text{H}}_{\text{1}}}-df_{{\text{H}}_{\text{0}}})\}/\{SSR_{{\text{H}}_{\text{1}}}/df_{{\text{H}}_{\text{1}}}\}$, where *SSR*_*H *_and *df*_*H *_denote the sum of squares and degrees of freedom, respectively, under the hypothesis H. The p-value can be calculated by a permutation distribution of the F statistic or an asymptotic distribution of the test statistic.

### 3) SAM-GS

SAM-GS extends SAM to gene-set analysis. SAM-GS tests a null hypothesis that the mean vectors of expression of genes in a gene set does not differ by the phenotype of interest. The SAM-GS method is based on individual t-like statistics from SAM, addressing the small variability problem encountered in microarray data, i.e., reducing the statistical significance associated with genes with very little variation in their expression. For each gene *j*, the *d *statistic is calculated as in SAM:

$$d(j)=\frac{{\bar{x}}_{1}(j)-{\bar{x}}_{2}(j)}{s(j)+s_{0}},$$

where the 'gene-specific scatter' *s*(*j*) is a pooled standard deviation over the two groups of the phenotype, and *s*_0 _is a small positive constant that adjusts for the small variability [1]. SAM-GS then summarizes these standardized differences in all genes in the gene set *S *by:

$$SAMGS=\sum_{i=1}^{\left|S\right|}d_{i}^{2}$$

A permutation distribution of the *SAMGS *statistic is used to calculate the p-value. We note that even though the recalculation of *s*_0 _is needed for each permutation, practically the implication is small, and both SAM and SAM-GS excel add-ins do not recalculate *s*_0_.

Each of the three methods provides a statistically valid test of the null hypothesis of no differential gene expression across a binary phenotype.

For the purpose of methodological comparisons, we also applied three "competitive null hypothesis" approaches to the analysis of the *p53 *dataset: Gene Set Enrichment Analysis (GSEA) [2]; the Significance Analysis of Function and Expression (SAFE) [16]; and Fisher's exact test [17]. Both GSEA and SAFE employ a two-stage approach to access the significance of a gene set. First, gene-specific measures are calculated that capture the association between expression and the phenotype of interest. Then a test statistic is constructed as a function of the gene-specific measures used in the first step. The significance of the test statistics is assessed by permutation of the response values. For GSEA, the Pearson correlation is used in the first step, according to Mootha *et al. *[2] and the Enriched Score is used in the second step. For SAFE, the student t-statistic is used in the first step and the Wilcoxon rank-sum test is used in the second step, both of these being the default options. For the Fisher's exact test, the list of significant genes is obtained from SAM [1]. An FDR cutoff of 0.3 assigned significance to 5% of the genes in the entire gene list.

## Availability and requirements

Project name: Comparison of statistical methods for gene set analysis based on testing self-contained hypotheses via. subject sampling.

Project home page: 

Operating system(s): Microsoft Windows XP

Programming language: R 2.4.x and Microsoft Excel 2003 or 2007

## Abbreviations

Significance Analysis of Microarray for Gene Sets (SAM-GS)

## Authors' contributions

JDP provided biological interpretations of the analysis results of the real-world dataset. QL and ID contributed significantly to data analysis, refinement of SAM-GS, and programming. The manuscript was written primarily by QL, ID, and YY, and critically reviewed and revised by all authors. All authors read and approved the final manuscript.

## Supplementary Material

Additional file 1 The analysis results of the two real-world microarray datasets (gender and leukemia) by the three methods. These three methods were applied and compared on two real-world microarray datasets: the male vs. female lymphoblastoid cell microarray dataset and the ALL- and AML-cell microarray dataset. Click here for file

Additional file 2 FDR values for the 17 gene sets listed in Table 2. FDR values of the 17 gene sets listed in Table 2 are presented. Click here for file

Additional file 3 P-values and FDR values for the three "self-contained null hypothesis" and three "competitive null hypothesis" approaches. The three "self-contained null hypothesis" and three "competitive null hypothesis" approaches were applied to the *p53 *dataset. The p-values and FDR values for the 17 gene sets listed in Table 2 are presented. Click here for file

## References

[B1] Tusher VG, Tibshirani R, Chu G (2001). Significance analysis of microarrays applied to the ionizing radiation response. Proc Natl Acad Sci USA.

[B2] Mootha VK, Lindgren CM, Eriksson KF, Subramanian A, Sihag S, Lehar J, Puigserver P, Carlsson E, Ridderstrale M, Laurila E, Houstis N, Daly MJ, Patterson N, Mesirov JP, Golub TR, Tamayo P, Spiegelman B, Lander ES, Hirschhorn JN, Altshuler D, Groop LC (2003). PGC-1alpha-responsive genes involved in oxidative phosphorylation are coordinately downregulated in human diabetes. Nat Genet.

[B3] Subramanian A, Tamayo P, Mootha VK, Mukherjee S, Ebert BL, Gillette MA, Paulovich A, Pomeroy SL, Golub TR, Lander ES, Mesirov JP (2005). Gene set enrichment analysis: a knowledge-based approach for interpreting genome-wide expression profiles. Proc Natl Acad Sci USA.

[B4] Goeman JJ, van de Geer SA, de Kort F, van Houwelingen HC (2004). A global test for groups of genes: testing association with a clinical outcome. Bioinformatics.

[B5] Goeman JJ, Oosting J, Cleton-Jansen AM, Anninga JK, van Houwelingen HC (2005). Testing association of a pathway with survival using gene expression data. Bioinformatics.

[B6] Mansmann U, Meister R (2005). Testing differential gene expression in functional groups. Goeman's global test versus an ANCOVA approach. Methods Inf Med.

[B7] Dinu I, Potter JD, Mueller T, Liu Q, Adewale AJ, Jhangri GS, Einecke G, Famulski KS, Halloran P, Yasui Y (2007). Improving GSEA for Analysis of Biologic Pathways for Differential Gene Expression across a Binary Phenotype. BMC Bioinformatics.

[B8] Goeman JJ, Bühlmann P (2007). Analyzing gene expression data in terms of gene sets: methodological issues. Bioinformatics.

[B9] Gene Set Enrichment Analysis. http://www.broad.mit.edu/gsea.

[B10] Huber W, von Heydebreck A, Sultmann H, Poustka A, Vingron M (2002). Variance stabilization applied to microarray data calibration and to the quantification of differential expression. Bioinformatics.

[B11] Huber W, von Heydebreck A, Sueltmann H, Poustka A, Vingron M (2003). Parameter estimation for the calibration and variance stabilization of microarray data. Stat Appl Genet Mol Biol.

[B12] Goeman JJ, Van de Geer SA, van Houwelingen HC (2006). Testing against a high dimensional alternative. J R Statist Soc B.

[B13] Storey JD (2002). A direct approach to false discovery rates. Journal of the Royal Statistical Society: Series B (Statistical Methodology).

[B14] Tian L, Greenberg SA, Kong SW, Altschuler J, Kohane IS, Park PJ (2005). Discovering statistically significant pathways in expression profiling studies. Proc Natl Acad Sci USA.

[B15] Tomfohr J, Lu J, Kepler TB (2005). Pathway level analysis of gene expression using singular value decomposition. BMC Bioinformatics.

[B16] Barry WT, Nobel AB, Wright FA (2005). Significance analysis of functional categories in gene expression studies: a structured permutation approach. Bioinformatics.

[B17] Draghici S, Khatri P, Martins RP, Ostermeier GC, Krawetz SA (2003). Global functional profiling of gene expression. Genomics.

[B18] le Cessie S, van Houwelingen HC (1995). Testing the fit of a regression model via score tests in random effects models. Biometrics.

[B19] Houwing-Duistermaat JJ, Derkx BH, Rosendaal FR, van Houwelingen HC (1995). Testing familial aggregation. Biometrics.

