# Supplementary material for: Comparative evaluation of gene-set analysis methods
Source: BMC Bioinformatics. 2007 Nov 7;8:431. doi: 10.1186/1471-2105-8-431 (PMC2238724; doi:10.1186/1471-2105-8-431)
Supplement: Additional file 1 — The analysis results of the two real-world microarray datasets (gender and leukemia) by the three methods. These three methods were applied and compared on two real-world microarray datasets: the male vs. female lymphoblastoid cell microarray dataset and the ALL- and AML-cell microarray dataset. [file 1471-2105-8-431-S1.pdf]

The analysis results of the two real-world microarray datasets (gender and leukemia) by the three methods

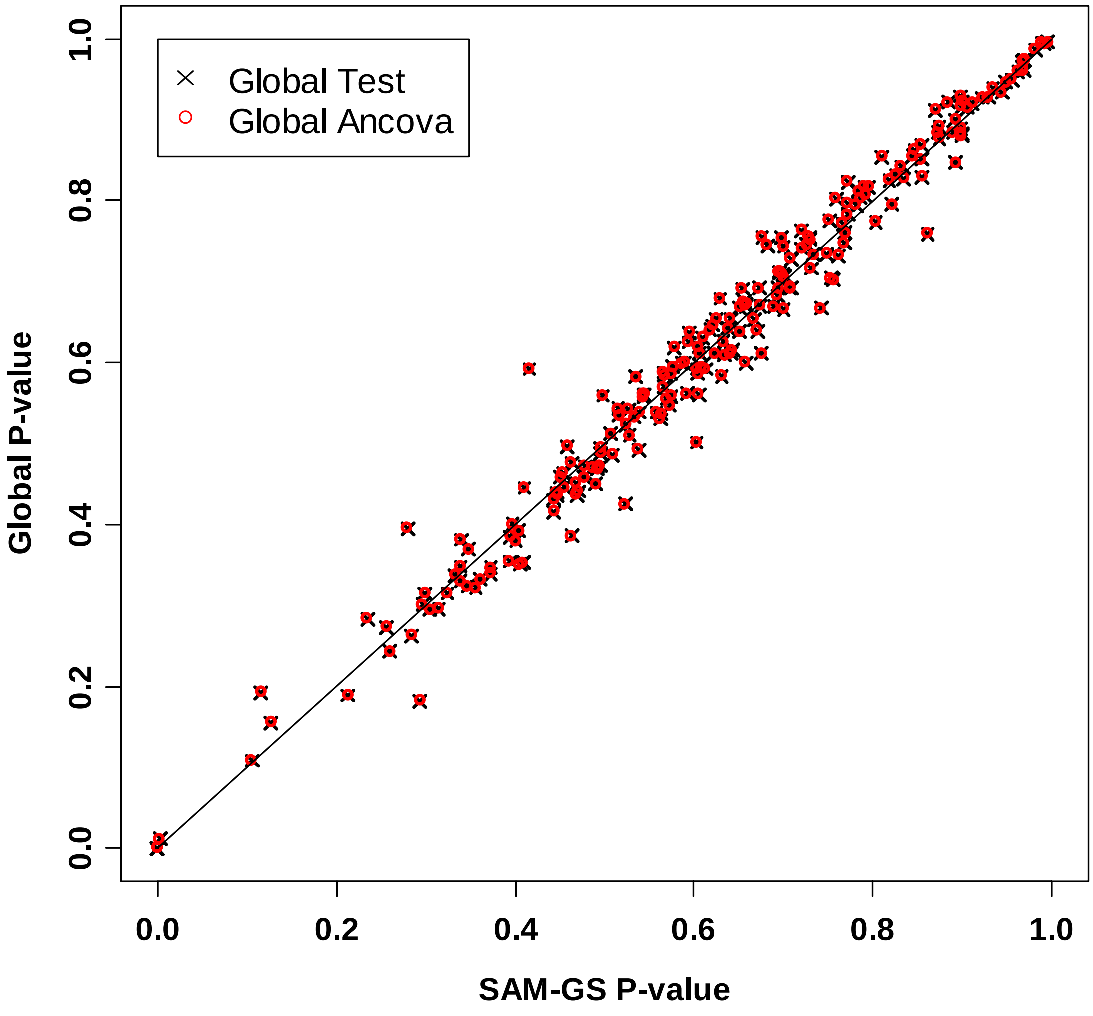

Figure S1. P-values of 212 gene sets in the Gender (C1) analysis: Global p-values after standardization vs. SAM-GS p-values before standardization. The line indicates equal p-values between SAM-GS and Global tests.

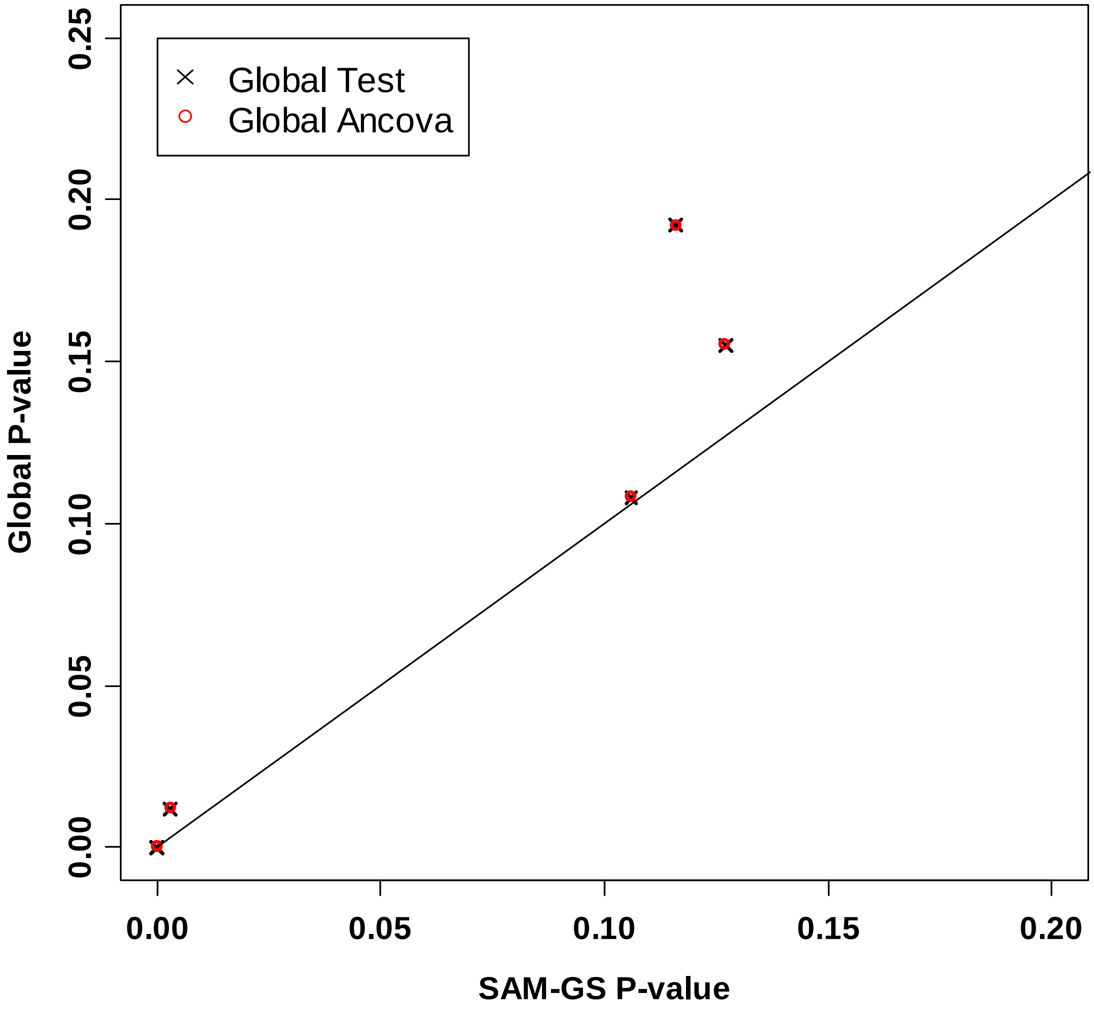

Figure S2: Lowest P-values in the Gender(C1) analysis: Global p-values after standardization vs. SAM-GS p-values before standardization. The line indicates equal p-values between SAM-GS and Global tests.

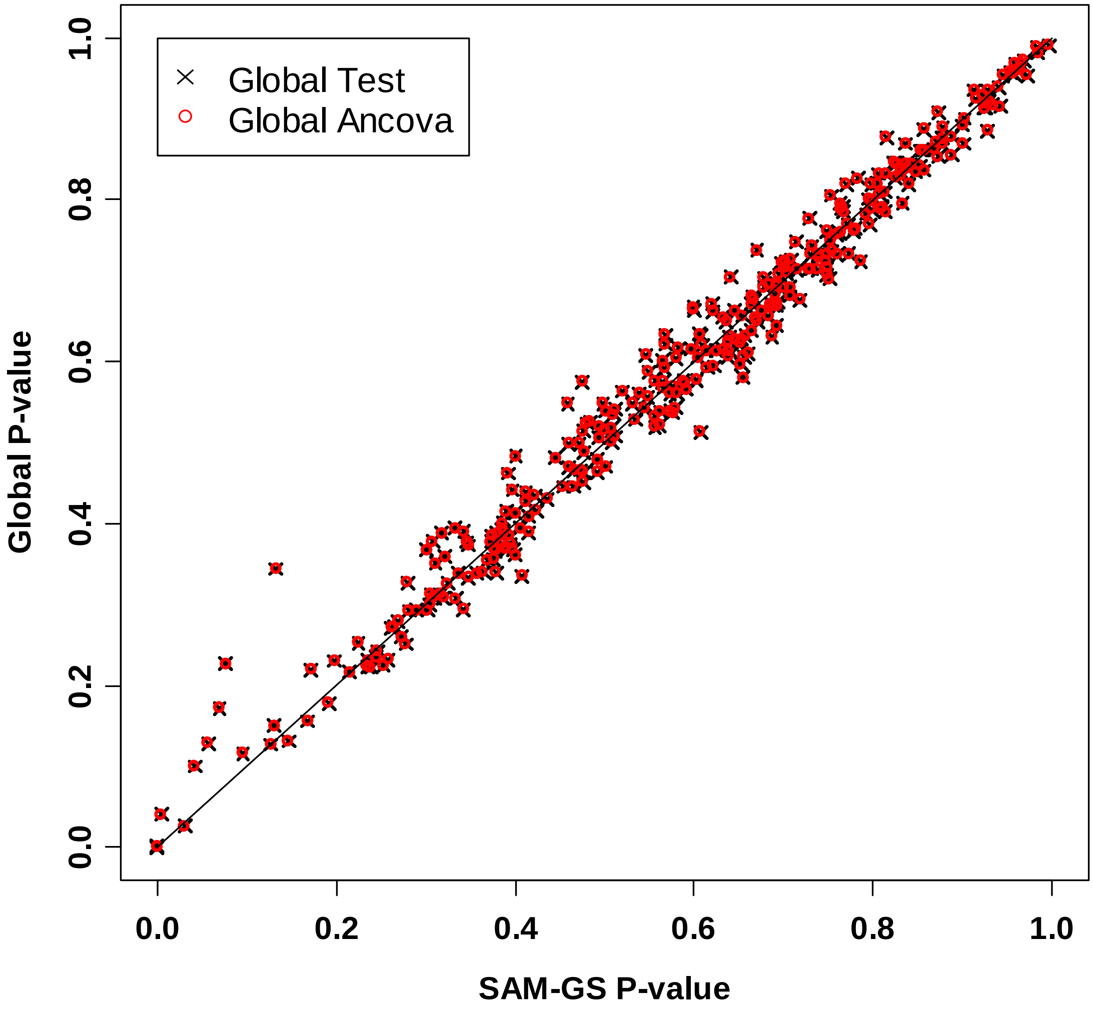

Figure S3. P-values of 318 gene sets in the Gender (C2) analysis: Global p-values after standardization vs. SAM-GS p-values before standardization. The line indicates equal p-values between SAM-GS and Global tests.

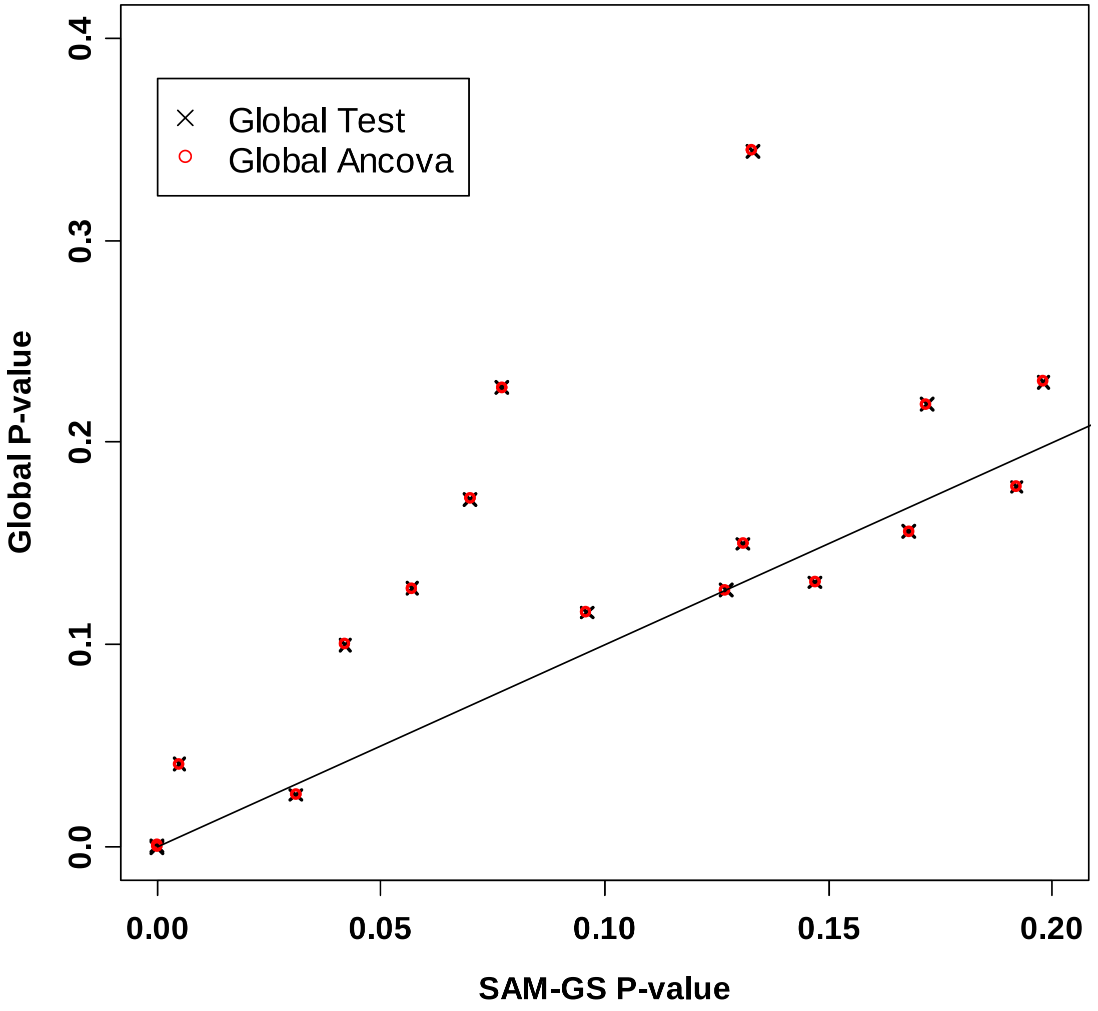

Figure S4: Lowest P-values in the Gender (C2) analysis: Global p-values after standardization vs. SAM-GS p-values before standardization. The line indicates equal p-values between SAM-GS and Global tests.
