# Supplementary material for: Comparative evaluation of gene-set analysis methods
Source: BMC Bioinformatics. 2007 Nov 7;8:431. doi: 10.1186/1471-2105-8-431 (PMC2238724; doi:10.1186/1471-2105-8-431)
Supplement: Additional file 2 — FDR values for the 17 gene sets listed in Table 2. FDR values of the 17 gene sets listed in Table 2 are presented. [file 1471-2105-8-431-S2.pdf]

FDR values for the 17 gene sets listed in table 2

Gene Set	Before standardization			After standardization			VSN		
	**Global**	**Ancova**	**SAM-GS**	**Global**	**Ancova**	**SAM-GS**	**Global**	**Ancova**	**SAM-GS**
ATM Pathway*	<0.0001	<0.0001	<0.0001	<0.0001	0.0036	<0.0001	0.0107	0.0057	<0.0001
BAD Pathway**	<0.0001	0.2112	<0.0001	<0.0001	<0.0001	<0.0001	0.0114	0.0133	<0.0001
Calcineurin Pathway$	0.5115	0.5194	<0.0001	0.0071	0.0036	<0.0001	0.0114	0.0133	0.0096
Cell cycle regulator†	0.3127	0.2992	<0.0001	0.0037	0.0026	<0.0001	0.0114	<0.0001	0.0047
Hsp27Pathway**	0.5115	0.5194	<0.0001	<0.0001	0.0026	<0.0001	0.0236	0.0133	<0.0001
Mitochondria pathway**	0.0834	0.2112	<0.0001	0.0071	0.0071	<0.0001	0.0265	0.0149	<0.0001
p53 signaling pathway*	0.5115	0.5194	<0.0001	0.0051	0.0046	0.0001	0.0161	0.0133	0.0069
*P53*_UP*	0.1042	0.2112	<0.0001	<0.0001	<0.0001	<0.0001	0.0302	0.0276	<0.0001
*p53*hypoxiaPathway*	0.5810	0.5890	<0.0001	<0.0001	<0.0001	<0.0001	0.0589	0.0511	<0.0001
*p53*Pathway*	0.5115	0.5194	<0.0001	<0.0001	<0.0001	<0.0001	<0.0001	0.0057	<0.0001
Raccycd Pathway†	0.5115	0.5194	<0.0001	0.0020	<0.0001	<0.0001	0.0114	0.0210	0.0069
Radiation_sensitivity*	0.5115	0.5194	<0.0001	<0.0001	<0.0001	<0.0001	0.0273	0.0295	<0.0001
SA_TRKA_RECEPTOR‡	0.5125	0.5226	<0.0001	0.0020	<0.0001	<0.0001	0.0114	0.0057	0.0069
bcl2family & reg. network**	0.5115	0.5194	0.0014	0.0020	0.0066	<0.0001	0.0226	0.0254	0.0018
Cell cycle arrest†	0.5115	0.5194	0.0014	0.0137	0.0113	0.0003	0.0114	0.0133	0.0077
Ceramide Pathway**	0.0834	0.2112	0.0014	0.0054	0.0058	<0.0001	0.0107	0.0057	<0.0001
CR_DEATH*	0.0695	0.2112	0.0063	0.0140	0.0113	0.0003	0.0918	0.0849	0.0069

* pathway member

** apoptosis

$ *p53*-induced proline oxidase mediates apoptosis via a calcineurin-dependent pathway

† cell cycle

‡ integrated negative feedback loop between Akt and *p53*
