# Supplementary material for: Comparative evaluation of gene-set analysis methods
Source: BMC Bioinformatics. 2007 Nov 7;8:431. doi: 10.1186/1471-2105-8-431 (PMC2238724; doi:10.1186/1471-2105-8-431)
Supplement: Additional file 3 — P-values and FDR values for the three "self-contained null hypothesis" and three "competitive null hypothesis" approaches. The three "self-contained null hypothesis" and three "competitive null hypothesis" approaches were applied to the p53 dataset. The p-values and FDR values for the 17 gene sets listed in Table 2 are presented. [file 1471-2105-8-431-S3.pdf]

P-values and FDR values for the three "self-contained null hypothesis" and three "competitive null hypothesis" approaches

P-values for the genes sets with P-value<=0.001 by any of the three "self-contained null hypothesis" in the analysis of the p53 dataset

Gene Set	Self-contained null hypothesis			Competitive null hypothesis		
	Global	Ancova	SAM-GS	SAFE#	GSEA#	Fisher#
ATM Pathway*	<0.001	0.002	<0.001	0.494	0.215	0.984
BAD Pathway**	<0.001	<0.001	<0.001	0.029	0.044	0.996
Calcineurin Pathway$	0.007	0.002	<0.001	0.668	0.138	0.933
Cell cycle regulator†	0.002	0.001	<0.001	0.025	0.293	0.969
Hsp27Pathway**	<0.001	0.001	<0.001	0.027	<0.001	1.000
Mitochondria pathway**	0.007	0.007	<0.001	0.543	0.329	0.923
p53 signaling pathway*	0.003	0.003	0.001	0.289	0.013	0.994
*P53*_UP*	<0.001	<0.001	<0.001	0.413	<0.001	1.000
*p53*hypoxiaPathway*	<0.001	<0.001	<0.001	0.343	<0.001	1.000
*p53*Pathway*	<0.001	<0.001	<0.001	0.273	<0.001	1.000
Raccycd Pathway†	0.001	<0.001	<0.001	0.117	0.565	0.891
Radiation_sensitivity*	<0.001	<0.001	<0.001	0.204	0.002	0.998
SA_TRKA_RECEPTOR‡	0.001	<0.001	<0.001	0.362	0.347	0.792
bcl2family & reg. network**	0.001	0.005	<0.001	0.064	0.426	0.880
Cell cycle arrest†	0.027	0.018	0.005	0.074	0.491	0.521
Ceramide Pathway**	0.004	0.004	<0.001	0.421	0.308	0.891
CR_DEATH*	0.029	0.017	0.004	0.718	0.314	0.833

* pathway member
** apoptosis
$ p53-induced proline oxidase mediates apoptosis via a calcineurin-dependent pathway
† cell cycle
‡ integrated negative feedback loop between Akt and p53
# the only additional gene set identified with p < 0.001 by any of SAFE, GSEA, and Fisher was HUMAN_CD34_ENRICHED_TF_JP. For this gene set, Fisher p-value was <0.001, but all the other five methods gave p-values > 0.37.

FDR values for the genes sets with P-value<=0.001 by any of the three

"self-contained null hypothesis" in the analysis of the p53 dataset

**Gene Set**	**Self-contained null hypothesis**			**Competitive null hypothesis**		
	**Global**	**Ancova**	**SAM-GS**	**SAFE**	**GSEA**	**Fisher**
ATM Pathway*	<0.0001	0.0036	<0.0001	0.5039	0.8734	1.0000
BAD Pathway**	<0.0001	<0.0001	<0.0001	0.4726	0.5750	1.0000
Calcineurin Pathway$	0.0071	0.0036	<0.0001	0.5168	0.8424	1.0000
Cell cycle regulator†	0.0037	0.0026	<0.0001	0.4726	0.9066	1.0000
Hsp27Pathway**	<0.0001	0.0026	<0.0001	0.4726	<0.0001	1.0000
Mitochondria pathway**	0.0071	0.0071	<0.0001	0.5039	0.8860	1.0000
p53 signaling pathway*	0.0051	0.0046	<0.0001	0.5039	0.5132	1.0000
*P53*_UP*	<0.0001	<0.0001	<0.0001	0.5039	0.0131	1.0000
*p53*hypoxiaPathway*	<0.0001	<0.0001	<0.0001	0.5039	<0.0001	1.0000
*p53*Pathway*	<0.0001	<0.0001	<0.0001	0.5039	<0.0001	1.0000
Raccycd Pathway†	0.0020	<0.0001	<0.0001	0.4972	0.8373	1.0000
Radiation_sensitivity*	<0.0001	<0.0001	<0.0001	0.4972	0.0778	1.0000
SA_TRKA_RECEPTOR‡	0.0020	<0.0001	<0.0001	0.5039	0.8359	1.0000
bcl2family & reg. network**	0.0020	0.0066	0.0014	0.4920	0.8377	1.0000
Cell cycle arrest†	0.0137	0.0113	0.0014	0.4920	0.9810	1.0000
Ceramide Pathway**	0.0054	0.0058	0.0014	0.5039	0.8894	1.0000
CR_DEATH*	0.0140	0.0113	0.0063	0.5304	0.8615	1.0000

* pathway member
** apoptosis
$ p53-induced proline oxidase mediates apoptosis via a calcineurin-dependent pathway
† cell cycle
‡ integrated negative feedback loop between Akt and p53
